# The Effectiveness of Low Dead Space Syringes for Reducing the Risk of Hepatitis C Virus Acquisition Among People Who Inject Drugs: Findings From a National Survey in England, Wales, and Northern Ireland^[Author-notes ciac140-FM1]^

**DOI:** 10.1093/cid/ciac140

**Published:** 2022-02-20

**Authors:** Adam Trickey, Sara Croxford, Eva Emanuel, Samreen Ijaz, Matthew Hickman, Joanna Kesten, Clare Thomas, Claire Edmundson, Monica Desai, Peter Vickerman

**Affiliations:** Population Health Sciences, University of Bristol, Bristol, United Kingdom; UK Health Security Agency, London, United Kingdom; UK Health Security Agency, London, United Kingdom; UK Health Security Agency, London, United Kingdom; Population Health Sciences, University of Bristol, Bristol, United Kingdom; NIHR Health Protection Research Unit in Behavioural Science and Evaluation at University of Bristol, Bristol, United Kingdom; Population Health Sciences, University of Bristol, Bristol, United Kingdom; NIHR Health Protection Research Unit in Behavioural Science and Evaluation at University of Bristol, Bristol, United Kingdom; The National Institute for Health Research Applied Research Collaboration West (NIHR ARC West) at University Hospitals Bristol and Weston NHS Foundation Trust, United Kingdom; Population Health Sciences, University of Bristol, Bristol, United Kingdom; NIHR Health Protection Research Unit in Behavioural Science and Evaluation at University of Bristol, Bristol, United Kingdom; The National Institute for Health Research Applied Research Collaboration West (NIHR ARC West) at University Hospitals Bristol and Weston NHS Foundation Trust, United Kingdom; UK Health Security Agency, London, United Kingdom; UK Health Security Agency, London, United Kingdom; Population Health Sciences, University of Bristol, Bristol, United Kingdom; NIHR Health Protection Research Unit in Behavioural Science and Evaluation at University of Bristol, Bristol, United Kingdom

**Keywords:** low dead space syringes, high dead space syringes, injecting drugs, IDU, HCV

## Abstract

Syringes with attached needles (termed fixed low dead space syringes [LDSS]) retain less blood following injection than syringes with detachable needles, but evidence on them reducing blood-borne virus transmission among people who inject drugs (PWID) is lacking. Utilizing the UK Unlinked Anonymous Monitoring cross-sectional bio-behavioral surveys among PWID for 2016/18/19 (n = 1429), we showed that always using fixed LDSS was associated with 76% lower likelihood (adjusted odds ratio  = 0.24, 95% confidence interval [CI]: .08–.67) of recent hepatitis C virus infection (RNA-positive and antibody-negative) among antibody-negative PWID compared to using any syringes with detachable needles.

Hepatitis C virus (HCV) is a bloodborne virus that heavily affects people who inject drugs (PWID) [1]. The primary interventions for preventing HCV transmission among PWID are needle and syringe programs (NSP) and opioid substitution therapy (OST) [2].

PWID either use syringes with fixed or detachable needles. Syringes with fixed needles are traditionally termed low dead space syringes (fixed LDSS) because their design minimizes the amount of dead or residual space between the syringe hub and needle when the plunger is fully depressed [3, 4]. Conversely, traditional syringes with detachable needles have greater dead space and are termed high dead space syringes (HDSS). Recent modifications to these syringes have reduced their dead space and are denoted detachable LDSS. Laboratory studies suggest that fixed LDSS transfer less virus than detachable LDSS and HDSS when re-used, while detachable LDSS transfer less virus than HDSS [4, 5]. Epidemiological studies suggest lower human immunodeficiency virus (HIV) and HCV prevalence among PWID that use fixed LDSS compared to those that use HDSS [6–8]. No studies have evaluated whether use of LDSS is associated with reduced incident infection.

The World Health Organization (WHO) [9] recommend that NSPs provide and encourage the use of LDSS by PWID. However, fixed LDSS only come in a limited range of volumes and needle gauges, with studies showing that PWID prefer greater variety to meet their differing needs [6, 10, 11]. Some PWID also prefer detachable needles so they can be swapped during an injecting episode if it becomes blunt [10]. This preference for syringes with detachable needles led to the development of detachable LDSS, with numerous settings expanding their distribution [12] to minimize the risks associated with using syringes with detachable needles. Our recent UK cost-effectiveness analysis suggested that this strategy could be cost-saving [13].

This analysis tests the hypothesis that using syringes with less dead space could reduce the risk of HCV acquisition.

## METHODS

### Data

This analysis focusses on the association between usage of fixed LDSS and the risk of recent HCV infection. We utilized the Unlinked Anonymous Monitoring (UAM) Survey, an annual cross-sectional bio-behavioral survey of people who have ever injected psychoactive drugs recruited from specialist harm reduction services across England, Wales, and Northern Ireland; the UAM Survey has been described elsewhere [14].

Those who participate in the survey completed a questionnaire about their drug use behaviors and demographics and provided a dried blood spot (DBS) sample that was tested for HCV antibodies (anti-HCV). From 2016, DBS samples that tested negative for anti-HCV were also tested for HCV RNA [14], indicating a recent primary HCV infection. Further details are in Supplementary Materials.

Participants were included in this analysis if they reported injecting in the past month, tested antibody-negative, and had an RNA test result. For each participant, we calculated the self-reported percentage of syringes used in the past month that had either detachable or attached/fixed needles (details in Supplementary Materials); excluding participants that received no needles. A binary variable was created for PWID that received 100% fixed LDSS (full use of syringes with fixed needles) or < 100% fixed LDSS (any use of syringes with detachable needles). We used multiple imputation by chained equations to account for missing data in covariates or the fixed LDSS variable, using 25 imputed data sets.

### Statistical Methods

We used logistic regression to estimate the unadjusted and adjusted association of 100% fixed LDSS use with recent primary HCV infection compared to < 100% fixed LDSS use. Variables assessed for inclusion in the adjusted model were pre-selected based on our previous analysis of associations of LDSS use with HCV prevalence (see Supplementary Materials) [6].

### Ethics

The UAM Survey has longstanding multisite ethics approval from London Research Ethics Committee (98/2/051) and the UK Health Security Agency (UKHSA: previously Public Health England).

## RESULTS

### Demographics and Injecting Characteristics

We included 1031 participants with information on type of syringe used in past month and 434 with imputed values for the fixed LDSS variable, giving 1465 participants in total. Of these 63.8% always used fixed LDSS, 25.5% always used syringes with detachable needles, and 10.7% used both.

Among 1465 PWID analyzed (Table 1), 92.4% had injected heroin in the past month, and 46.9% had injected crack. The mean age was 37.3 years, 26.2% were female, and duration of injecting was 13.0 years. There were 33 (2.3%) recent primary HCV infections (antibody-negative participants testing RNA-positive) in the sample.

**Table 1.** Sociodemographic and Injecting Characteristics of People Who Inject Drugs (PWID) for the Sample Data Set by Whether They Used Fixed Low Dead Space Syringes (LDSS) 100% of the Time or Not

Variable	0–99% Use of Fixed LDSS^a^	100% Use of Fixed LDSS^a,b^	Total
No. (%)	530 (36.2%)	935 (63.8%)	1465 (100.0%)
Mean (95% CI)			
Age (years)	37.5 (36.8–38.2)	37.1 (36.6–37.7)	37.3 (36.9–37.7)
Duration of injecting (years)	14.0 (13.3–14.7)	12.4 (11.8–13.0)	13.0 (12.5–13.4)
Number of injections in last month	33.9 (31.7–36.0)	31.8 (30.0–33.5)	32.5 (31.1–33.9)
Percentage (95% CI)			
Female	26.4% (22.8–30.2%)	26.1% (23.4–29.1%)	26.2% (24.0–28.5%)
Injected heroin in last month	93.0% (90.6–94.9%)	92.2% (90.2–93.7%)	92.4% (91.1–93.8%)
Injected crack in last month	51.3% (47.1–55.5%)	44.5% (41.3–47.7%)	46.9% (44.3–49.5%)
Injected in groin in last month	55.7% (51.5–59.8%)	13.5% (11.4–15.9%)	28.9% (26.5–31.2%)
Ever incarcerated	63.0% (58.9–67.0%)	54.8% (51.6–58.0%)	57.8% (55.3–60.4%)
Currently homeless	29.1% (25.5–33.1%)	33.1% (30.1–36.2%)	33.1% (30.2–36.1%)
Currently have 100% NSP coverage	61.0% (56.8–65.0%)	61.0% (57.8–64.2%)	60.9% (57.9–64.0%)
Currently on OST	76.2% (72.4–79.6%)	63.1% (59.9–66.2%)	67.9% (65.5–70.3%)
Shared any injecting equipment last month	16.1% (13.3–19.5%)	19.9% (17.5–22.6%)	18.1% (16.1–20.1%)
Region			
East of England	7.9% (5.9–10.5%)	7.9% (6.4–9.9%)	7.9% (6.5–9.3%)
London	7.9% (5.9–10.5%)	8.4% (6.8–10.4%)	8.2% (6.8–9.6%)
Southeast England	12.6% (10.1–15.7%)	14.8% (12.6–17.2%)	14.0% (12.2–15.8%)
Southwest England	11.1% (8.8–14.1%)	12.2% (10.2-14.5%)	11.8% (10.2–13.5%)
West Midlands of England	11.4% (8.9–14.3%)	12.6% (10.6–14.9%)	12.2% (10.5–13.8%)
Northwest England	6.6% (4.8–9.0%)	7.7% (6.2–9.6%)	7.3% (6.0–8.6%)
Yorkshire and Humberside	8.6% (6.5–11.3%)	6.9% (5.4–8.7%)	7.5% (6.2–8.9%)
East Midlands of England	13.6% (10.9–16.7%)	7.5% (6.0–9.4%)	9.8% (8.2–11.3%)
Northeast England	9.5% (7.3–12.3%)	9.7% (7.9–11.8%)	9.6% (8.1–11.1%)
Wales	7.7% (5.7–10.3%)	10.2% (8.4–12.4%)	9.3% (7.8–10.8%)
Northern Ireland	3.1% (1.9–5.0%)	2.1% (1.3–3.2%)	2.5% (1.7–3.3%)
Year of survey			
2016	33.7% (29.8–37.8%)	32.2% (29.3–35.3%)	32.8% (30.4–35.2%)
2018	29.7% (26.0–33.6%)	31.6% (28.6–34.6%)	30.9% (28.5–33.2%)
2019	36.6% (32.7–40.8%)	36.2% (33.2–39.4%)	36.4% (33.9–38.8%)
Markers of recent infection (RNA-positive and antibody negative)	3.8% (2.5–5.8%)	1.3% (0.7–2.3%)	2.3% (1.5–3.0%)

Abbreviations: CI, confidence interval; NSP, needle and syringe programs; OST, opioid substitution therapy.

Characteristics and behaviors for these subgroups were calculated for the first of the 25 imputed data sets, as due to the changing denominators across these data sets, the mean across the 25 imputed data sets was not computable.

Any use of syringes with detachable needles.

Characteristics of the PWID always using fixed LDSS and those using any syringes with detachable needles were similar, except that fewer in the 100% fixed LDSS group had injected into the groin (13.5% vs 55.7%).

### LDSS Use and Risk of HCV Acquisition

Over the whole sample (for the first imputed data set), there were fewer recent HCV infections among individuals always using fixed LDSS (1.3%; 95% confidence interval [CI]: .7–2.3%) than among individuals using any syringes with detachable needles (3.8%; 95% CI: 2.5–5.8%). These percentages were similar in a complete case analysis.

Compared to any use of syringes with detachable needles, exclusive use of fixed LDSS was associated with lower odds of having recent HCV infection (Table 2, adjusted odds ratio [aOR] .24; 95% CI .08–0.67, *P* = .007). The only other variable associated with recent infection was injecting crack in the past month (aOR 3.09; 95% CI 1.24–7.69). The association between LDSS use and recent HCV infection was slightly attenuated if imputation was not used: aOR 0.31 (95% CI: .12–.81, *P* = .016). Although the odds ratios (ORs) for other variables remained consistent between the univariable and multivariable analyses, the OR for injecting in the groin went from 1.16 (95% CI: .54–2.47) to 0.59 (95% CI: .24–1.47).

**Table 2.** Unadjusted and Mutually Adjusted Odds Ratios (OR) with 95% Confidence Intervals (95% CI) of Recent Hepatitis C Virus (HCV) Infection

Variable	Unadjusted OR (95% CI)	Adjusted OR (95% CI)	Adjusted *P* Value
0–99% use of fixed LDSS^a^	1	1	
100% use of fixed LDSS	0.32 (.14–.74)	0.24 (.08–.67)	.007
Male	1	1	
Female	2.11 (1.05–4.26)	1.96 (.88–4.35)	.100
Injecting duration: 0–4 years	1	1	
5–9 years	1.01 (.38–2.64)	1.17 (.41–3.33)	.764
10–14 years	0.38 (.10–1.38)	0.40 (.10–1.58)	.226
15 + years	0.53 (.23–1.23)	0.58 (.22–1.54)	.275
Not injecting heroin	1	1	
Injecting heroin	2.65 (.36–19.60)	1.84 (.22–15.56)	.577
Not injecting crack	1	1	
Injecting crack	3.09 (1.43–6.70)	3.09 (1.24–7.69)	.016
Not injecting in groin	1	1	
Injecting in groin	1.16 (.54–2.47)	0.59 (.24–1.47)	.255
<100% NSP coverage	1	1	
≥100% NSP coverage	1.37 (.62–3.03)	1.92 (.69–5.32)	.212
Never imprisoned	1	1	
Ever imprisoned	1.19 (.58–2.44)	1.46 (.64–3.37)	.371
Not currently homeless	1	1	
Currently homeless	1.79 (.83–3.85)	1.46 (.64–3.34)	.374
N injections per month: 0–9	1	1	
10–19	1.34 (.43–4.19)	1.31 (.36–4.78)	.678
20–29	0.35 (.03–2.91)	0.32 (.03–3.18)	.331
30–49	0.77 (.15–3.85)	0.86 (.15–5.01)	.864
≥50	1.32 (.52–3.33)	1.36 (.41–4.52)	.615
Not shared injecting equipment	1	1	
Shared injecting equipment	1.83 (.84–4.01)	1.78 (.74–4.28)	.197
Not currently on OST	1	1	
Currently on OST	0.60 (.29–1.21)	0.58 (.26–1.30)	.189
Region: East of England	1	1	
London	0.97 (.06–15.63)	1.06 (.06–18.21)	.966
Southeast	3.47 (.41–29.15)	3.06 (.34–27.52)	.318
Southwest	5.58 (.69–45.18)	4.96 (.57–43.24)	.147
West Midlands	1.31 (.12–14.57)	1.38 (.12–16.48)	.798
Northwest	6.83 (.81–57.70)	6.66 (.74–60.11)	.091
Yorkshire and Humber	2.13 (.19–23.82)	3.14 (.26–38.06)	.369
East Midlands	1.63 (.15–18.21)	1.61 (.13–19.41)	.708
Northeast	3.36 (.37–30.45)	5.04 (.51–49.93)	.167
Wales	0.85 (.05–13.77)	0.91 (.05–15.75)	.949
Northern Ireland	NA	NA	.993
Survey year: 2016	1	1	
2018	1.15 (.52–2.56)	0.91 (.37–2.24)	.831
2019	0.59 (.24–1.47)	0.52 (.19–1.40)	0.198

Abbreviations: LDSS, low dead space syringes; NA, not available: perfect predictor of failure/success; NSP, needle and syringe provision; OST, opiate substitution therapy.

Any use of syringes with detachable needles.

## DISCUSSION

Our analysis shows for the first time that exclusive use of low dead space syringes with attached needles (fixed LDSS) could be associated with reduced risk of HCV acquisition among PWID compared to using syringes with detachable needles.

### Comparison With Other Studies

Our study are consistent with and builds on previous laboratory studies [4, 5, 13] by producing the first empirical estimate for the effectiveness of using fixed LDSS to reduce the risk of HCV acquisition. Consistent with this study, previous studies have found that injecting crack or other stimulants is associated with heightened HCV incidence [14, 15]. Recent systematic reviews have found that currently being on OST or high coverage NSP can reduce HCV acquisition risk [2], although incarceration [16] or homelessness [17] can increase HCV acquisition risk. Our study findings broadly agree with these systematic reviews, although our results lack power. The only exception is high coverage NSP where our study suggests no association with reduced HCV risk [14].

### Strengths and Limitations

Our analysis’s main strength was that we could assess whether the use of syringes with fixed or detachable needles was associated with recent incident HCV infection, however, there were limitations. First, we used a marker of recent infection instead of the gold standard for incidence studies of using longitudinal follow-up for identifying new infections. The short window period associated with this marker means only 33 incident infections were identified. This dependence on few incident infections emphasizes the importance of replicating the study in other settings. Additionally, using a marker of recent infection means there could be some misclassification of recent infections, although previous studies suggest this should be small [18]. Our analysis depended on self-reported data for all behavioral and intervention related factors, which may bias some variables, such as sharing of injecting equipment due to stigma associated with this behavior. This bias could mean that the association of injecting equipment sharing with incident HCV infection may be masked in this dataset. This is unlikely to explain the lower risk of HCV infection associated with using fixed LDSS because fixed LDSS use is associated with greater equipment sharing (19.9% vs 16.1%). Our analysis was also limited by using a variable that could only distinguish between syringes with fixed or detachable needles. This meant we could only assess whether using syringes with attached needles (fixed LDSS) was associated with reduced infection risk. This is still crucial information because it suggests that syringe dead space is an important determinant of infectivity. Many survey participants also did not complete all the questions needed to create the LDSS variable. This meant that we relied on imputed data in our main analysis; however, associations were similar when we did not use imputed data set. Due to the observational nature of our study we cannot rule out confounding factors that may be associated with both the risk of HCV acquisition and use of LDSS. Controlling for a wide range of potential confounders minimizes this risk. Our sample was mostly heroin injectors who had been injecting for over a decade, which may limit its generalizability to younger injecting cohorts or those predominantly using stimulants.

Our analysis did not consider whether use of LDSS reduces the risk of HIV acquisition; data are needed on this.

## IMPLICATIONS AND CONCLUSIONS

That the use of fixed LDSS is associated with a large reduction in an individual’s risk of HCV acquisition suggests that a syringe’s dead space is an important determinant of its infectivity. We encourage further studies to collect data on LDSS exposure to corroborate our findings, ideally with longitudinal follow-up. Nonetheless, given this evidence and our cost-effectiveness data [13], programs should encourage PWID to use fixed LDSS to minimize their risk of acquiring HIV and HCV infection, and provide syringes with detachable needles that minimize the dead space associated with that type of syringe. These findings have global implications because they suggest NSPs should focus on how they minimize the dead space of syringes that they distribute, while still meeting the varying syringe needs of PWID [6, 10, 11]. Although there are now many different syringe options that attempt to minimize the dead space of syringes with detachable needles (detachable LDSS), studies suggest that some have greater dead space than others [4]. It is therefore important that different types of detachable LDSS are evaluated using standard methods to determine their dead space and to assess their acceptability for PWID [10]. This needs to feed into international guidance on the best syringes for NSPs to use for improving their effectiveness, something that is important for achieving HCV and HIV elimination among PWID. These changes need to occur in parallel to increases in NSP coverage, which is currently low globally [19].

## Supplementary Data

Supplementary materials are available at Clinical Infectious Diseases online. Consisting of data provided by the authors to benefit the reader, the posted materials are not copyedited and are the sole responsibility of the authors, so questions or comments should be addressed to the corresponding author.

ciac140_suppl_Supplementary_Material

## Notes

***Author contributions.*** P. V. and A. T. had the original concept for the study and developed the analysis plan. A. T. performed the analyses with input from P. V. and S. C.; P. V. wrote the first draft of the article with input from A. T. Also, S. C., E. E., S. I., M. D., and C. E. collected, provided, and verified the data. A. T., M. D., M. H., J. K., C. T., S. C., E. E., C. E., and  P. V. contributed to data interpretation, writing the report, and approved the final version.

***Acknowledgments.*** This study was partially funded by the National Institute for Health Research Health Protection Research Units (NIHR HPRUs) in Behavioural Science and Evaluation at the University of Bristol in partnership with the UK Health Security Agency (UKHSA—previously Public Health England). P. V. is also supported by the Wellcome Trust and the US National Institute of Allergy and Infectious Diseases and National Institute for Drug Abuse (NIAID/NIDA grant numbers R01AI147490, R01DA033679, R21 DA047902). A. T. is funded through the Wellcome Trust (222770/Z/21/Z). J. K. and C. T. are partly funded by National Institute for Health Research Applied Research Collaboration West (NIHR ARC West) and NIHR Health Protection Research Unit in Behavioral Science and Evaluation.

***Disclaimer.*** The views expressed in this publication are those of the authors and not necessarily those of the National Health Service, the NIHR, the Department of Health and Social Care, or the UKHSA.

***Financial support.*** This study was partially funded by the National Institute for Health Research Health Protection Research Units (NIHR HPRUs) in Behavioral Science and Evaluation at the University of Bristol in partnership with the UK Health Security Agency (UKHSA—previously Public Health England).
