# Supplementary material for: The Effectiveness of Low Dead Space Syringes for Reducing the Risk of Hepatitis C Virus Acquisition Among People Who Inject Drugs: Findings From a National Survey in England, Wales, and Northern Ireland
Source: Clin Infect Dis. 2022 Feb 20;75(6):1073–7. doi: 10.1093/cid/ciac140 (PMC9522423; doi:10.1093/cid/ciac140)
Supplement: ciac140_suppl_Supplementary_Material [file ciac140_suppl_supplementary_material.docx]

**The effectiveness of low dead space syringes for reducing the risk of hepatitis C virus acquisition among people who inject drugs - findings from a national survey in England, Wales, and Northern Ireland**

Adam Trickey^1^, Sara Croxford^2^, Eva Emanuel^2^, Samreen Ijaz^2^, Matthew Hickman^1,3^, Jo Kesten^1,3,4^, Clare Thomas^1,3,4^, Claire Edmundson^2^, Monica Desai^2^, Peter Vickerman^1,3^

1 Population Health Sciences, University of Bristol, Bristol, UK

2 UK Health Security Agency, London, UK

3 NIHR Health Protection Research Unit in Behavioural Science and Evaluation at University of Bristol, Bristol, UK

4 The National Institute for Health Research Applied Research Collaboration West (NIHR ARC West) at University Hospitals Bristol and Weston NHS Foundation Trust, UK

**Introduction**

Hepatitis C virus (HCV) is a bloodborne virus with considerable burden globally. Nearly half of HCV infections worldwide are thought to be due to injecting drug use(1), with over half of people who inject drugs (PWID) having been infected(2). The primary interventions for preventing HCV transmission among PWID are needle and syringe programmes (NSP) and opioid substitution therapy (OST), which have high coverage (>50%) in the UK(3) but low coverage globally(4). Evidence suggests these interventions can reduce the risk of HCV acquisition(3).

PWID either use syringes with fixed or detachable needles. Syringes with fixed needles are traditionally termed low dead space syringes (fixed LDSS) because their design minimises the amount of dead or residual space between the syringe hub and needle (volume of space in which fluid is retained) when the plunger is fully depressed(5, 6). In contrast, traditional syringes with detachable needles have much greater dead space and are termed high dead space syringes (HDSS). Recent modifications to these syringes have reduced their dead space and are denoted detachable LDSS. Existing laboratory studies suggest that if fixed LDSS are re-used they will transfer less virus than detachable LDSS and HDSS, while detachable LDSS transfer less virus than HDSS (7)(6, 8). Modelling supports this laboratory evidence(9, 10), estimating that smaller quantities of blood are transferred with fixed LDSS than HDSS. Limited epidemiological studies also suggest lower HIV and HCV prevalence among PWID that use fixed LDSS compared to those that use HDSS(11-14), but no studies have evaluated whether use of LDSS is associated with reduced incident infection.

The World Health Organization (WHO)(15) and UK National Institute for Health and Care Excellence(16) recommend that NSPs provide and encourage the use of LDSS by PWID. However, fixed LDSS only come in a limited range of volumes and needle gauges, with studies showing that PWID prefer a wider range of sizes and needle lengths to enable them to inject different quantities of drugs or to inject into veins that require longer needles(11, 17, 18). Some PWID prefer detachable needles so they can be swapped during an injecting episode if it becomes blunt due to them having difficulty locating a vein(17). This preference for syringes with detachable needles led to the development of detachable LDSS, with Wales, Scotland (from 2016), and some regions of England recently expanding the distribution of these syringes to minimize the risks associated with using syringes with detachable needles. Similarly, limited data show that other countries are expanding the use of detachable LDSS(19, 20). A recent UK cost-effectiveness analysis suggested that this strategy is likely to be cost saving if detachable LDSS reduce the risk of HCV acquisition by just 0.26% compared to HDSS(21).

The WHO and UNAIDS have set targets for elimination of HCV and HIV by 2030(22, 23). There is therefore an urgent need for empirical evidence on the effectiveness of new interventions, such as use of LDSS, to help guide policy on how best to reduce the incidence of HCV and HIV among PWID, and so reach elimination. This analysis uses data from an annual cross-sectional survey among PWID in England, Northern Ireland, and Wales, to test the hypothesis that using syringes with less dead space could reduce the risk of HCV acquisition as measured through a marker of recent infection.

**Methods**

*Data*

These analyses build on our previous study looking at the association between usage of syringes with fixed needles (fixed LDSS) and HCV prevalence(11). This analysis focusses on the association between usage of fixed LDSS and the risk of recent HCV infection. We used data from the Unlinked Anonymous Monitoring (UAM) Survey, an annual bio-behavioural survey of people who have ever injected psychoactive drugs recruited from specialist services providing drug treatment, NSPs, and outreach work across England, Wales, and Northern Ireland; the UAM Survey has been described in detail previously(24).

Those who agreed to participate were asked to self-complete a short questionnaire about their drug use behaviours and demographics and provide a dried blood spot (DBS) sample that was tested for HCV antibodies (anti-HCV), in addition to antibodies for HIV and hepatitis B virus. From 2016, DBS sampled from the UAM Survey that tested negative for anti-HCV have also been routinely tested for HCV RNA, indicating a recent primary HCV infection. Methods for how the RNA testing was undertaken on DBS samples have been described previously(25). Previous studies show that this is a reliable measure of recent primary infection, with little misclassification(26). However, the time period that someone remains in this state after infection (the ‘window period’) is short (probably <2 months(26)), so large sample sizes and/or high incidence rates are required to study these events. We were unable to determine if antibody positive individuals had experienced reinfections and so this analysis only considers primary incident infections. Questions on the use of syringes with fixed needles (fixed LDSS) or detachable needles were included from 2014 onwards, except for 2017, and so this study focuses on data from 2016, 2018, and 2019, which have data on both the use of fixed LDSS and recent infection.

Participants were included in these analyses if they reported injecting in the past month and had an HCV antibody and RNA test result. Only those participants that were antibody negative were retained, removing those with positive antibody tests or insufficient tests. For each participant, we calculated the percentage of syringes used in the past month that had either detachable or attached/fixed needles from the questions “How many individual needles (including ones attached to syringes) did you get from Needle Exchanges during the last month (28 days)?” and “How many of these needles were already attached to syringes (barrels)?”. Syringes with detachable needles will include some new detachable LDSS, which have become more common over the survey years considered. Although these syringes reduce the dead space associated with syringes with detachable needles (~55-88%), evidence suggests their dead space is still 4-15 times greater than fixed LDSS(20). Unfortunately, we were unable to determine use of this type of syringe from the questions included in the survey. Needles received from NSPs should cover almost all needles received in the UK, as public health funded NSPs are widely available throughout the UK, and over the counter sales of needles are restricted. We excluded participants that received no needles in the previous month. A binary variable was created for PWID that received 100% fixed LDSS (full use of syringes with fixed needles) or <100% fixed LDSS (any use of syringes with detachable needles). We used multiple imputation by chained equations to account for missing data in covariates or the fixed LDSS usage variable, using 25 imputed datasets.

*Statistical Methods*

Our previous analysis considered which characteristics of PWID were associated with use of fixed LDSS(11), and so this was not considered in detail here. We used logistic regression to estimate the unadjusted and adjusted association of 100% fixed LDSS use, compared to any use of syringes with detachable needles, with recent HCV infection (being RNA-positive and antibody negative). Variables assessed for inclusion in the adjusted model were pre-selected based on previous analyses of associations with HCV prevalence. These variables were sex, region of recruitment, calendar year, years since first injection, groin injecting in last month, injecting crack in the last month, injecting heroin in the last month, NSP coverage (binary based on whether enough syringes were collected to cover all their injections or not), currently being on OST, any equipment or syringe sharing (whether LDSS or HDSS) in last month, injection frequency (number of days injected last month multiplied by the frequency of injecting on last day they injected, and then split into categories), having ever been to prison, and current homelessness.

*Ethics*

The UAM Survey has longstanding multisite ethics approval from London Research Ethics Committee (98/2/051) and the UK Health Security Agency (UKHSA: previously Public Health England). This is a secondary analysis of anonymised data and so further ethics consideration is not required.

**Results**

*Demographics and injecting characteristics*

Of 4,473 surveyed individuals who had injected in the previous month, 2,981 (67%) were excluded because they were HCV sero-positive; 27 (2%) of those remaining were excluded as they reported having received no needles in the last month. Of those still included, 434 (29.6%) had missing information that prevented creation of the fixed LDSS usage variable, and so their fixed LDSS usage was imputed. Of those 1,031 participants with information on what type of syringe they used over the past month, 63.7% always used fixed LDSS, 25.3% always used syringes with detachable needles, and 10.9% used both. When we also included those with imputed values for the fixed LDSS variable, giving 1,465 in total, 63.8% always used fixed LDSS, 25.5% always used syringes with detachable needles and 10.7% used both.

Amongst 1,465 PWID analysed (table 1), 92.4% had injected heroin in the past month and 46.9% had injected crack. The mean age was 37.3 years and 26.2% were female. The mean number of injections in the previous month was 32.5 and the mean time since first injection was 13.0 years. Overall, 57.8% had ever been incarcerated, 33.1% were currently homeless, 60.9% currently collected enough syringes from NSP to cover their injections, 18.1% reported any equipment or syringe sharing in the past month, 28.9% injected in the groin in the last month, and 67.9% reported currently being on OST. There were 33 (2.3%) recent incident infections (antibody negative participants testing RNA-positive) in the sample.

Characteristics of the PWID using fixed LDSS all the time and those using syringes with detachable needles some of the time were similar, except that fewer in the 100% fixed LDSS group had ever been incarcerated (54.8% vs 63.0%), were currently on OST (63.1% vs 76.2%), or injected into the groin (13.5% vs 55.7%).

*LDSS use and risk of HCV acquisition*

Over the whole sample (including those with imputed data for the first imputed dataset), there were fewer recent HCV infections among individuals using fixed LDSS (1.3%; 95%CI 0.7-2.3%) in the last month than among individuals using any syringes with detachable needles (3.8%; 95%CI 2.5-5.8%). In a complete case analysis excluding missing data, these percentages were 1.3% (95%CI: 0.7-2.6%) and 3.7% (95%CI: 2.2-6.2%), respectively.

Compared to any use of syringes with detachable needles, exclusive use of fixed LDSS during the last month was associated with lower odds of having recent HCV infection (table 2, OR 0.32; 95%CI 0.14-0.74, p=0.008). The only other variable associated with recent HCV infection at the univariate level was injecting crack in the past month (OR 3.09; 95%CI 1.43-6.70). In the adjusted model, only injecting crack was associated with increased odds of recent HCV infection (aOR 3.09, 95%CI 1.24-7.69), whilst exclusive LDSS use remained associated with lower odds of recent HCV infection (aOR 0.24; 95%CI 0.08-0.67, p=0.007). This association held but was slightly attenuated if imputation was not used to assign an LDSS status to the 434 PWID with insufficient data: unadjusted OR 0.36 (95%CI 0.15-0.83, p=0.017), aOR 0.31; (95%CI 0.12-0.81, p=0.016). Whilst the ORs for the other variables remained relatively consistent between the univariable and multivariable analyses, the OR for injecting in the groin went from 1.16 (95%CI 0.54-2.47) to 0.59 (95%CI: 0.24-1.47).

**Discussion**

Our analysis shows for the first time that exclusive use of low dead space syringes with attached needles (fixed LDSS) could be associated with a reduction in the risk of HCV acquisition among PWID compared to using syringes with detachable needles. Additionally, PWID that inject into their groin are far less likely to always use fixed LDSS syringes, as has been noted previously(11) because these syringes do not have sufficiently long needles for injecting in the groin(17).

*Comparison with other studies*

Previous studies have undertaken laboratory or modelling analyses to determine whether LDSS are likely to transfer less HIV or HCV than HDSS when shared(6), while epidemiological studies have shown that use of LDSS can be associated with reduced HIV or HCV prevalence among PWID(11, 13, 14). When applying the findings from previous laboratory studies(6) to a UK setting in our recent cost-effectiveness analysis(21), our model estimated that usage of fixed LDSS may result in a 73.5% (95% Credibility Interval 63.3–81.5%) risk reduction of becoming infected compared to using traditional HDSS, which is consistent with our empirical estimates from this study. Our study builds on these previous studies by producing the first empirical estimate for the effectiveness of using fixed LDSS to reduce the risk of HCV acquisition.

Other studies have also considered predictors of HCV incidence among PWID(3, 27-29), with some of these studies being from the UK and using the UAM Survey(24, 25, 30). As in this study, previous studies have found that crack injecting or injecting of stimulants(27) is associated with heightened HIV or HCV incidence, including in the UK(25, 30). Recent systematic reviews have found that currently being on OST or high coverage NSP can reduce HCV acquisition risk(3), while ever or recently being incarcerated(29) or recently being homeless(28) can increase HCV acquisition risk. Our study findings broadly agree with these systematic reviews, although our results lack power. The only exception is for high coverage NSP where our study suggests no tendency for it being associated with reduced HCV risk, similar to a previous analysis involving the UAM Survey(25).

*Strengths and limitations*

Our analysis’s main strength was that we could assess whether the use of syringe with fixed or detachable needles was associated with recent incident HCV infection, however, there were several limitations. First, we used a marker of recent infection instead of the gold standard for incidence studies of using longitudinal follow-up for identifying new infections. The short window period associated with this marker means only 33 incident infections were identified. This dependence on a low number of incident infections means it is important that this study is replicated in other datasets to confirm our findings in other settings. This low power also meant we could not undertake more detailed analyses to consider whether regions solely using detachable LDSS (Wales from 2016) had a reduced association between using syringes with fixed or detachable needles and the risk of HCV acquisition. Additionally, using a marker of recent infection means there may have been some misclassification of recent infections, although previous studies suggest this should be small(26). Second, our analysis depended on self-reported data for all behavioural and intervention related factors, which may bias some variables, such as sharing of injecting equipment where there is generally reluctance to report this behaviour due to stigma and fear of judgement. This bias could mean that the association of injecting equipment sharing with incident HCV infection may be masked in this dataset. This issue is unlikely to explain the lower risk of HCV infection associated with using fixed LDSS because use of fixed LDSS is associated with greater sharing of injecting equipment (19.9% vs 16.1%). Our analysis was also limited by using a simple variable that could only distinguish between syringes with fixed or detachable needles. This meant we could not distinguish between individuals using detachable LDSS or traditional HDSS and so our analysis could only assess whether using syringes with attached needles (fixed LDSS) was associated with reduced infection risk. This is still crucial information because it suggests that syringe dead space is an important determinant of infectivity, and so emphasises the necessity for strategies that reduce the dead space of syringes distributed by NSP. Many survey participants also did not complete all the questions needed to create the LDSS variable. This meant we relied on imputed values for this variable in our main analysis, however, associations were similar when we did not use imputed dataset. Due to the observational nature of our study we cannot rule out confounding factors that may be associated with both the risk of HCV acquisition and use of LDSS, although we are unsure what these could be. Controlling for a wide range of potential confounders strengthens our results. Our sample was mostly heroin injectors who had been injecting for over a decade, which is generalisable to injecting cohorts in most high-income countries, however, our results may not be generalisable to younger injecting cohorts or those predominantly using stimulants.

Our analysis did not consider whether use of LDSS reduces the risk of HIV acquisition. Although our findings should be relevant for other settings and for HIV because they provide evidence that syringe dead space is an important determinant of syringe infectivity, more data is needed to confirm our findings in other settings and for HIV, ideally with studies following up participants at multiple time points. As HIV incidence tends to be lower than HCV incidence among PWID, larger studies would be required to do this.

*Implications and conclusions*

That the use of fixed LDSS is associated with a large reduction in an individual’s risk of HCV acquisition, suggests that a syringe’s dead space is an important determinant of its infectivity. We encourage further studies, such as surveys of HCV re-infection to collect data on NSP and LDSS exposure to corroborate our findings. Nonetheless, given this evidence and our cost-effectiveness data(21), programmes should encourage PWID to use fixed LDSS to minimise their risk of HIV and HCV infection, and provide syringes with detachable needles that minimise the dead space associated with that type of syringe. These findings have global relevance for the design of NSPs for PWID because they suggest NSPs should focus on how they minimise the dead space of syringes that they distribute, while still meeting the varying syringe needs and preferences of different PWID(11, 17, 18). Although there are now many different syringe options that attempt to minimise the dead space of syringes with detachable needles (i.e., detachable LDSS), studies suggest that some have greater dead space than others(6). It is therefore important that different types of detachable LDSS are evaluated using standard methods to determine their dead space and to assess their acceptability for PWID(17). This needs to feed into international guidance on the best syringes for programmes to use to minimise their dead space and to meet the varying needs of PWID. This could potentially improve the effectiveness of existing NSPs, which we have shown to be cost saving(21), and is likely to be important for achieving HCV and HIV elimination among PWID. However, to achieve this, it is also important that these changes occur in parallel to increases in NSP coverage, which is currently very low globally(4).

**References**

1. Trickey A, Fraser H, Lim AG, Peacock A, Colledge S, Walker JG, et al. The contribution of injection drug use to hepatitis C virus transmission globally, regionally, and at country level: a modelling study. Lancet Gastroenterol Hepatol. 2019;4(6):435-44.

2. Degenhardt L, Peacock A, Colledge S, Leung J, Grebely J, Vickerman P, et al. Global prevalence of injecting drug use and sociodemographic characteristics and prevalence of HIV, HBV, and HCV in people who inject drugs: a multistage systematic review. Lancet Glob Health. 2017;5(12):e1192-e207.

3. Platt L, Minozzi S, Reed J, Vickerman P, Hagan H, French C, et al. Needle syringe programmes and opioid substitution therapy for preventing hepatitis C transmission in people who inject drugs. Cochrane Database Syst Rev. 2017;9:CD012021.

4. Larney S, Peacock A, Leung J, Colledge S, Hickman M, Vickerman P, et al. Global, regional, and country-level coverage of interventions to prevent and manage HIV and hepatitis C among people who inject drugs: a systematic review. Lancet Glob Health. 2017;5(12):e1208-e20.

5. Zule WA. Low dead-space syringes for preventing HIV among people who inject drugs: promise and barriers. Current opinion in HIV and AIDS. 2012;7(4):369-75.

6. Binka M, Paintsil E, Patel A, Lindenbach BD, Heimer R. Survival of Hepatitis C Virus in Syringes Is Dependent on the Design of the Syringe-Needle and Dead Space Volume. PLoS One. 2015;10(11):e0139737.

7. Zule WA, Cross HE, Stover J, Pretorius C. Are major reductions in new HIV infections possible with people who inject drugs? The case for low dead-space syringes in highly affected countries. Int J Drug Policy. 2013;24(1):1-7.

8. Paintsil E, He H, Peters C, Lindenbach BD, Heimer R. Survival of hepatitis C virus in syringes: implication for transmission among injection drug users. J Infect Dis. 2010;202(7):984-90.

9. Vickerman P, Martin NK, Hickman M. Could low dead-space syringes really reduce HIV transmission to low levels? Int J Drug Policy. 2013;24(1):8-14.

10. Zule WA, Ticknor-Stellato KM, Desmond DP, Vogtsberger KN. Evaluation of needle and syringe combinations. J Acquir Immune Defic Syndr Hum Retrovirol. 1997;14(3):294-5.

11. Trickey A, May MT, Hope V, Ward Z, Desai M, Heinsbroek E, et al. Usage of low dead space syringes and association with hepatitis C prevalence amongst people who inject drugs in the UK. Drug and alcohol dependence. 2018;192:118-24.

12. Zule WA, Oramasionwu C, Evon D, Hino S, Doherty IA, Bobashev GV, et al. Event-level analyses of sex-risk and injection-risk behaviors among nonmedical prescription opioid users. The American journal of drug and alcohol abuse. 2016;42(6):689-97.

13. Zule WA, Bobashev G. High dead-space syringes and the risk of HIV and HCV infection among injecting drug users. Drug and alcohol dependence. 2009;100(3):204-13.

14. Zule WA, Desmond DP, Neff JA. Syringe type and drug injector risk for HIV infection: a case study in Texas. Soc Sci Med. 2002;55(7):1103-13.

15. Walsh N, Verster A, Rodolph M, Akl EA. WHO guidance on the prevention of viral hepatitis B and C among people who inject drugs. Int J Drug Policy. 2014;25(3):363-71.

16. National Institute for Health and Care Excellence. ICE Guideline PH52. Needle and syringe programmes [<https://www.nice.org.uk/guidance/ph52/chapter/1-recommendations>]. 2014.

17. Kesten JM, Ayres R, Neale J, Clark J, Vickerman P, Hickman M, et al. Acceptability of low dead space syringes and implications for their introduction: A qualitative study in the West of England. Int J Drug Policy. 2017;39:99-108.

18. Zule WA, Latypov A, Otiashvili D, Kirtadze I, Ibragimov U, Bobashev GV. Factors that influence the characteristics of needles and syringes used by people who inject drugs in Tajikistan. Harm reduction journal. 2015;12:37.

19. Zule WA, Latypov A, Otiashvili D, Bangel S, Bobashev GV. Feasibility of needle and syringe programs in Tajikistan distributing low dead space needles. Harm reduction journal. 2018;15(1):44.

20. Zule WA, Pande PG, Otiashvili D, Bobashev GV, Friedman SR, Gyarmathy VA, et al. Options for reducing HIV transmission related to the dead space in needles and syringes. Harm reduction journal. 2018;15(1):3.

21. Hancock E, Ward Z, Ayres R, Neale J, Hussey D, Kesten JM, et al. Detachable low dead space syringes for the prevention of hepatitis C among people who inject drugs in Bristol, UK: an economic evaluation. Addiction. 2020;115(4):702-13.

22. World Health Organisation. Combating hepatitis B and C to reach elimination by 2030 (<http://www.who.int/hepatitis/publications/hep-elimination-by-2030-brief/en/>). 2016.

23. UNAIDS. Fast-Track - Ending the AIDS epidemic by 2030 (<https://www.unaids.org/en/resources/documents/2014/JC2686_WAD2014report>). Geneva; 2014.

24. Cullen KJ, Hope VD, Croxford S, Shute J, Ncube F, Parry JV. Factors associated with recently acquired hepatitis C virus infection in people who inject drugs in England, Wales and Northern Ireland: new findings from an unlinked anonymous monitoring survey. Epidemiol Infect. 2015;143(7):1398-407.

25. Hope VD, Harris RJ, Vickerman P, Platt L, Shute J, Cullen KJ, et al. A comparison of two biological markers of recent hepatitis C virus (HCV) infection: implications for the monitoring of interventions and strategies to reduce HCV transmission among people who inject drugs. Euro Surveill. 2018;23(47).

26. Page-Shafer K, Pappalardo BL, Tobler LH, Phelps BH, Edlin BR, Moss AR, et al. Testing strategy to identify cases of acute hepatitis C virus (HCV) infection and to project HCV incidence rates. J Clin Microbiol. 2008;46(2):499-506.

27. Morris MD, Shiboski S, Bruneau J, Hahn JA, Hellard M, Prins M, et al. Geographic Differences in Temporal Incidence Trends of Hepatitis C Virus Infection Among People Who Inject Drugs: The InC3 Collaboration. Clin Infect Dis. 2017;64(7):860-9.

28. Arum C, Fraser H, Artenie AA, Bivegete S, Trickey A, Alary M, et al. Homelessness, unstable housing, and risk of HIV and hepatitis C virus acquisition among people who inject drugs: a systematic review and meta-analysis. Lancet Public Health. 2021;6(5):e309-e23.

29. Stone J, Fraser H, Lim AG, Walker JG, Ward Z, MacGregor L, et al. Incarceration history and risk of HIV and hepatitis C virus acquisition among people who inject drugs: a systematic review and meta-analysis. The Lancet Infectious diseases. 2018;18(12):1397-409.

30. Platt L, Sweeney S, Ward Z, Guinness L, Hickman M, Hope V, et al. Assessing the impact and cost-effectiveness of needle and syringe provision and opioid substitution therapy on hepatitis C transmission among people who inject drugs in the UK: an analysis of pooled data sets and economic modelling. Public Health Research. 2017.

**Table 1**: Socio-demographic and injecting characteristics of people who inject drugs (PWID) for the sample dataset by whether they used fixed low dead space syringes (LDSS) 100% of the time or not.

| **Variable** | **0-99% use of fixed LDSS*** | **100% use of fixed LDSS*†** | **Total** |
| --- | --- | --- | --- |
| **Number (%)** | **530 (36.2%)** | **935 (63.8%)** | **1465 (100.0%)** |
| **Mean (95% confidence interval)** | | | |
| Age (years) | 37.5 (36.8-38.2) | 37.1 (36.6-37.7) | 37.3 (36.9-37.7) |
| Duration of injecting (years) | 14.0 (13.3-14.7) | 12.4 (11.8-13.0) | 13.0 (12.5-13.4) |
| Number of injections in last month | 33.9 (31.7-36.0) | 31.8 (30.0-33.5) | 32.5 (31.1-33.9) |
| **Percentage (95% confidence interval)** | | | |
| Female | 26.4% (22.8-30.2%) | 26.1% (23.4-29.1%) | 26.2% (24.0-28.5%) |
| Injected heroin in last month | 93.0% (90.6-94.9%) | 92.2% (90.2-93.7%) | 92.4% (91.1-93.8%) |
| Injected crack in last month | 51.3% (47.1-55.5%) | 44.5% (41.3-47.7%) | 46.9% (44.3-49.5%) |
| Injected in groin in last month | 55.7% (51.5-59.8%) | 13.5% (11.4-15.9%) | 28.9% (26.5-31.2%) |
| Ever incarcerated | 63.0% (58.9-67.0%) | 54.8% (51.6-58.0%) | 57.8% (55.3-60.4%) |
| Currently homeless | 29.1% (25.5-33.1%) | 33.1% (30.1-36.2%) | 33.1% (30.2-36.1%) |
| Currently have 100% NSP coverage | 61.0% (56.8-65.0%) | 61.0% (57.8-64.2%) | 60.9% (57.9-64.0%) |
| Currently on OST | 76.2% (72.4-79.6%) | 63.1% (59.9-66.2%) | 67.9% (65.5-70.3%) |
| Shared any injecting equipment last month | 16.1% (13.3-19.5%) | 19.9% (17.5-22.6%) | 18.1% (16.1-20.1%) |
| Region East of England | 7.9% (5.9-10.5%) | 7.9% (6.4-9.9%) | 7.9% (6.5-9.3%) |
| London | 7.9% (5.9-10.5%) | 8.4% (6.8-10.4%) | 8.2% (6.8-9.6%) |
| South East England | 12.6% (10.1-15.7%) | 14.8% (12.6-17.2%) | 14.0% (12.2-15.8%) |
| South West England | 11.1% (8.8-14.1%) | 12.2% (10.2-14.5%) | 11.8% (10.2-13.5%) |
| West Midlands of England | 11.4% (8.9-14.3%) | 12.6% (10.6-14.9%) | 12.2% (10.5-13.8%) |
| North West England | 6.6% (4.8-9.0%) | 7.7% (6.2-9.6%) | 7.3% (6.0-8.6%) |
| Yorkshire and Humberside | 8.6% (6.5-11.3%) | 6.9% (5.4-8.7%) | 7.5% (6.2-8.9%) |
| East Midlands of England | 13.6% (10.9-16.7%) | 7.5% (6.0-9.4%) | 9.8% (8.2-11.3%) |
| North East England | 9.5% (7.3-12.3%) | 9.7% (7.9-11.8%) | 9.6% (8.1-11.1%) |
| Wales | 7.7% (5.7-10.3%) | 10.2% (8.4-12.4%) | 9.3% (7.8-10.8%) |
| Northern Ireland | 3.1% (1.9-5.0%) | 2.1% (1.3-3.2%) | 2.5% (1.7-3.3%) |
| Year of survey 2016 | 33.7% (29.8-37.8%) | 32.2% (29.3-35.3%) | 32.8% (30.4-35.2%) |
| 2018 | 29.7% (26.0-33.6%) | 31.6% (28.6-34.6%) | 30.9% (28.5-33.2%) |
| 2019 | 36.6% (32.7-40.8%) | 36.2% (33.2-39.4%) | 36.4% (33.9-38.8%) |
| **Markers of recent infection (RNA-positive and antibody negative)** | **3.8% (2.5-5.8%)** | **1.3% (0.7-2.3%)** | **2.3% (1.5-3.0%)** |

*Characteristics and behaviours for these subgroups were calculated for the first of the 25 imputed datasets, as due to the changing denominators across these datasets, the mean across the 25 imputed datasets was not computable. **†** any use of syringes with detachable needles. NSP: Needle and syringe provision. OST: Opiate substitution therapy.

**Table 2**: Unadjusted and mutually adjusted odds ratios (OR) with 95% confidence intervals (95%CI) of recent hepatitis C virus (HCV) infection

| **Variable** | **Unadjusted OR (95%CI)** | **Adjusted OR (95%CI)** | **Adjusted p-value** |
| --- | --- | --- | --- |
| 0-99% use of fixed LDSS* | 1 | 1 |  |
| 100% use of fixed LDSS | 0.32 (0.14-0.74) | 0.24 (0.08-0.67) | 0.007 |
|  |  |  |  |
| Male | 1 | 1 |  |
| Female | 2.11 (1.05-4.26) | 1.96 (0.88-4.35) | 0.100 |
|  |  |  |  |
| Injecting duration: 0-4 years | 1 | 1 |  |
| 5-9 years | 1.01 (0.38-2.64) | 1.17 (0.41-3.33) | 0.764 |
| 10-14 years | 0.38 (0.10-1.38) | 0.40 (0.10-1.58) | 0.226 |
| 15+ years | 0.53 (0.23-1.23) | 0.58 (0.22-1.54) | 0.275 |
|  |  |  |  |
| Not injecting heroin | 1 | 1 |  |
| Injecting heroin | 2.65 (0.36-19.60) | 1.84 (0.22-15.56) | 0.577 |
|  |  |  |  |
| Not injecting crack | 1 | 1 |  |
| Injecting crack | 3.09 (1.43-6.70) | 3.09 (1.24-7.69) | 0.016 |
|  |  |  |  |
| Not injecting in groin | 1 | 1 |  |
| Injecting in groin | 1.16 (0.54-2.47) | 0.59 (0.24-1.47) | 0.255 |
|  |  |  |  |
| <100% NSP coverage | 1 | 1 |  |
| >=100% NSP coverage | 1.37 (0.62-3.03) | 1.92 (0.69-5.32) | 0.212 |
|  |  |  |  |
| Never imprisoned | 1 | 1 |  |
| Ever imprisoned | 1.19 (0.58-2.44) | 1.46 (0.64-3.37) | 0.371 |
|  |  |  |  |
| Not currently homeless | 1 | 1 |  |
| Currently homeless | 1.79 (0.83-3.85) | 1.46 (0.64-3.34) | 0.374 |
|  |  |  |  |
| N injections per month: 0-9 | 1 | 1 |  |
| 10-19 | 1.34 (0.43-4.19) | 1.31 (0.36-4.78) | 0.678 |
| 20-29 | 0.35 (0.03-2.91) | 0.32 (0.03-3.18) | 0.331 |
| 30-49 | 0.77 (0.15-3.85) | 0.86 (0.15-5.01) | 0.864 |
| 50+ | 1.32 (0.52-3.33) | 1.36 (0.41-4.52) | 0.615 |
|  |  |  |  |
| Not shared injecting equipment | 1 | 1 |  |
| Shared injecting equipment | 1.83 (0.84-4.01) | 1.78 (0.74-4.28) | 0.197 |
|  |  |  |  |
| Not currently on OST | 1 | 1 |  |
| Currently on OST | 0.60 (0.29-1.21) | 0.58 (0.26-1.30) | 0.189 |
|  |  |  |  |
| Region: East of England | 1 | 1 |  |
| London | 0.97 (0.06-15.63) | 1.06 (0.06-18.21) | 0.966 |
| South East | 3.47 (0.41-29.15) | 3.06 (0.34-27.52) | 0.318 |
| South West | 5.58 (0.69-45.18) | 4.96 (0.57-43.24) | 0.147 |
| West Midlands | 1.31 (0.12-14.57) | 1.38 (0.12-16.48) | 0.798 |
| North West | 6.83 (0.81-57.70) | 6.66 (0.74-60.11) | 0.091 |
| Yorkshire & Humber | 2.13 (0.19-23.82) | 3.14 (0.26-38.06) | 0.369 |
| East Midlands | 1.63 (0.15-18.21) | 1.61 (0.13-19.41) | 0.708 |
| North East | 3.36 (0.37-30.45) | 5.04 (0.51-49.93) | 0.167 |
| Wales | 0.85 (0.05-13.77) | 0.91 (0.05-15.75) | 0.949 |
| Northern Ireland | NA | NA | 0.993 |
|  |  |  |  |
| Survey year: 2016 | 1 | 1 |  |
| 2018 | 1.15 (0.52-2.56) | 0.91 (0.37-2.24) | 0.831 |
| 2019 | 0.59 (0.24-1.47) | 0.52 (0.19-1.40) | 0.198 |

LDSS: Low dead space syringes. NA: Not available: perfect predictor of failure/success; * any use of syringes with detachable needles. OST: Opiate substitution therapy. NSP: Needle and syringe provision.

**Figure 1:** Odds ratios (95% confidence intervals) for risk of hepatitis C virus (HCV) acquisition comparing 100% use of fixed low dead space syringes (LDSS) to any use of syringes with detachable needles (0-99% use of fixed LDSS) across various analyses
